# Biomolecular environment, quantification, and intracellular interaction of multifunctional magnetic SERS nanoprobes†
†Electronic supplementary information (ESI) available. See DOI: 10.1039/c6an00890a



**DOI:** 10.1039/c6an00890a

**Published:** 2016-06-29

**Authors:** Tina Büchner, Daniela Drescher, Virginia Merk, Heike Traub, Peter Guttmann, Stephan Werner, Norbert Jakubowski, Gerd Schneider, Janina Kneipp

**Affiliations:** a Humboldt-Universität zu Berlin , Department of Chemistry , Brook-Taylor-Str. 2 , 12489 Berlin , Germany . Email: janina.kneipp@chemie.hu-berlin.de; b Humboldt-Universität zu Berlin , School of Analytical Sciences Adlershof (SALSA) , Albert-Einstein-Str. 5-9 , 12489 Berlin , Germany; c BAM Federal Institute for Materials Research and Testing , Richard-Willstätter-Str. 11 , 12489 Berlin , Germany; d Helmholtz-Zentrum Berlin für Materialien und Energie , BESSY II , Albert-Einstein-Str. 15 , 12489 Berlin , Germany

## Abstract

Multifunctional composite nanoprobes consisting of iron oxide nanoparticles linked to silver and gold nanoparticles, Ag–Magnetite and Au–Magnetite, respectively, were introduced by endocytic uptake into cultured fibroblast cells. The cells containing the non-toxic nanoprobes were shown to be displaceable in an external magnetic field and can be manipulated in microfluidic channels. The distribution of the composite nanostructures that are contained in the endosomal system is discussed on the basis of surface-enhanced Raman scattering (SERS) mapping, quantitative laser ablation inductively coupled plasma mass spectrometry (LA-ICP-MS) micromapping, and cryo soft X-ray tomography (cryo soft-XRT). Cryo soft-XRT of intact, vitrified cells reveals that the composite nanoprobes form intra-endosomal aggregates. The nanoprobes provide SERS signals from the biomolecular composition of their surface in the endosomal environment. The SERS data indicate the high stability of the nanoprobes and of their plasmonic properties in the harsh environment of endosomes and lysosomes. The spectra point at the molecular composition at the surface of the Ag–Magnetite and Au–Magnetite nanostructures that is very similar to that of other composite structures, but different from the composition of pure silver and gold SERS nanoprobes used for intracellular investigations. As shown by the LA-ICP-MS data, the uptake efficiency of the magnetite composites is approximately two to three times higher than that of the pure gold and silver nanoparticles.

## Abstract 2

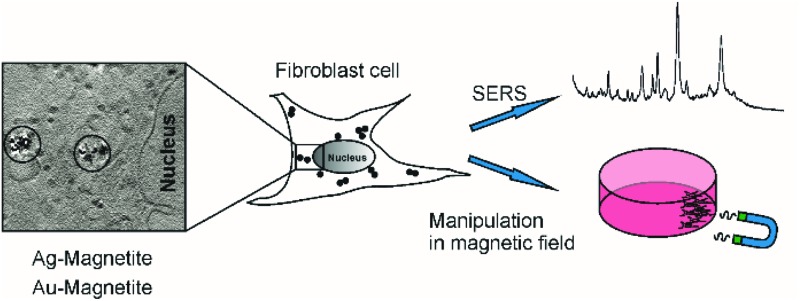
Multifunctional composite nanoprobes, Ag–Magnetite and Au–Magnetite, were manipulated in fibroblast cells and characterized using SERS, LA-ICP-MS, and nanotomography.

## Introduction

Magnetic nanoparticles of different materials have found widespread application in diagnostic imaging, therapeutics, and biotechnology. Specifically in the theranostic field, multifunctional nanomaterials, that in addition to serving as contrast agents in MRI^1,2^ allow for optical imaging and/or manipulation of biomaterials, such as the selective destruction of cancer cells by local heating, have become a subject of extensive research.^3–10^ The ability to move magnetic nanomaterials in magnetic fields provides new possibilities for the manipulation and transport of biological molecules,^11,12^ drugs,^13,14^ and live cells.^15,16^ Meanwhile, the magnetophoresis of live cells has found widespread application, ranging from basic quantification of nanomaterials^17^ to the detection and isolation of bacteria in water.^18,19^ The manipulation of whole eukaryotic cells using magnetic nanostructures is especially powerful in microfluidic structures, where local magnetic forces can be applied efficiently,^20,21^ and different cell types can be sorted,^22,23^ such as apoptotic cells^24^ or specific cancer cells.^25,26^ In order to assess the uptake mechanisms used by the cells,^17,27^ and to find out about physiological consequences^28^ and potential cytotoxicity^29^ during the modification of eukaryotic cells with magnetic nanomaterials, it is necessary to understand the interaction of magnetic nanostructures with the cellular biomolecules and their behavior in the cell.

Surface-enhanced Raman scattering (SERS) spectra of reporter or label molecules can be obtained from composite plasmonic nanostructures with magnetic properties.^30–35^ Spectra of magnetic SERS labels were also employed to visualize their interaction with cells, *e.g.*, when particles with plasmonic and magnetic properties bind to cell membranes,^36–38^ or when they enter a cell.^39,40^ In bacteria, plasmonic–magnetic nanostructures were also shown to provide SERS spectral information from the molecules in the cell walls of bacterial cells.^41–43^

In the work presented here, we study the interaction of composite magnetic–plasmonic nanoprobes (Ag–Magnetite and Au–Magnetite) inside eukaryotic cells by observing the molecular composition in the proximity of the composite structures by SERS. The nanostructures serve as multifunctional probes of their endosomal environment in cells that can be manipulated, *e.g.*, in microfluidic structures. The localization and distribution of the nanocomposites with the cellular ultrastructure is characterized by cryo soft X-ray nanotomography (cryo soft-XRT) in intact, vitrified cells. As we will discuss, their uptake and spatial distribution can be quantified by laser ablation inductively coupled plasma mass spectrometry (LA-ICP-MS).

## Experimental

### Nanoparticle synthesis and characterization

For all experiments, ultrapure water (18 MΩ) was used. Gold(iii)chloride trihydrate (99.9%), silver nitrate (99.9999%), hydroxylamine hydrochloride, sodium hydroxide, iron(ii)sulfate heptahydrate and iron(iii)chloride hexahydrate (97%) were purchased from Sigma-Aldrich Chemie GmbH (Taufkirchen, Germany) and trisodium citrate dihydrate (99%) from Merck (Darmstadt, Germany).

To obtain gold nanoparticles according to ref. 44, 250 mL of 1 mM gold(iii)chloride trihydrate solution was heated under reflux for 10 min before 25 mL of 40 mM trisodium citrate dihydrate solution was added and boiled for 10 min. The reaction mixture was stirred for 15 min at room temperature. The UV-vis spectrum of gold nanoparticles exhibits a plasmon band with a maximum at ∼530 nm.

For the preparation of silver nanoparticles,^45^ 80 μmol hydroxylamine hydrochloride was dissolved in 5 mL of 31.5 mM sodium hydroxide solution. A silver nitrate solution (50 μmol, 45 mL) was added and stirred for 30 minutes. A plasmon band with a maximum at ∼417 nm was observed in the spectrum of the silver nanoparticles.

Magnetite nanoparticles were obtained by dissolving 1 mmol iron(ii)sulfate heptahydrate and 2 mmol iron(iii)chloride hexahydrate in 1.6 mL of 0.4 M hydrochloric acid according to ref. 43. Then, 16.6 mL of 1.5 M sodium hydroxide solution was added, and the resulting black nanoparticles were separated using a grade N48 neodymium magnet. The nanoparticles were washed several times with water and hydrochloric acid.

For linking the magnetite nanoparticles to silver and gold nanoparticles in the composite nanostructures, a procedure described in ref. 46 was used. To a mixture of 210 μL of magnetite solution and 790 μL of water, 385 μmol (3-aminopropyl)triethoxysilane (APTES, 98%, ABCR GmbH & Co. KG, Germany) was added. After stirring for 6 hours, the nanoparticles were separated using a grade N48 neodymium magnet, washed several times with water and ethanol and diluted in 1.5 mL of water. Ag–Magnetite nanostructures were obtained by mixing silver nanoparticles and APTES-magnetite at a ratio (v : v) of 150 : 14, and Au–Magnetite by mixing gold nanoparticles and APTES-magnetite at a ratio of 1300 : 12. Both composite nanoparticle suspensions were stirred for 30 minutes, respectively, separated using a grade N48 neodymium magnet, and washed three times with water. Plasmon bands with maxima at 418 nm and 534 nm were found in the UV-vis spectra of Ag–Magnetite and Au–Magnetite (Fig. S1†), respectively.

Absorbance spectra of the nanoparticle suspensions were recorded using a V-670 double-beam ultraviolet–visible (UV–vis)/near-infrared (NIR) spectrophotometer (JASCO, Gross-Umstadt, Germany). The particle size was determined by transmission electron microscopy with a Tecnai G2 20S- TWIN microscope (FEI, Hillsboro, USA). Crystal violet (10^–6^ M) was used to determine the SERS enhancement factor of the composite nanoparticles as described in ref. 47, for an excitation wavelength of 785 nm.

### Cell culture

Cell culture media and phosphate-buffered saline (PBS) were purchased from Biochrom AG, Berlin, Germany. Swiss albino mouse fibroblast cells of the cell line 3T3 (DSMZ, Braunschweig, Germany) were cultured in Dulbecco's modified Eagle's medium (DMEM) with 10% fetal calf serum (FCS) and 1% ZellShield™ in a humidified environment at 37 °C and 5% CO_2_. For LA-ICP-MS and SERS experiments, 3T3 cells were grown as a monolayer on sterile cover-slips (Thermo Fisher Scientific, Schwerte, Germany) in a six-well plate and incubated with 1 mL of 10% Ag–Magnetite and Au–Magnetite suspension, respectively, in standard cell culture medium for 3 and 24 hours. For cryo soft X-ray tomography experiments, fibroblasts were grown on Formvar-coated grids under the same conditions. The cytotoxicity of Ag–Magnetite and Au–Magnetite was evaluated using the XTT reagent (2,3-bis-(2-methoxy-4-nitro-5-sulfophenyl)-2*H*-tetrazolium-5-carboxanilide) (Biozol Diagnostica, Eching, Germany) in a procedure described in ref. 48. To manipulate the cells in a magnetic field, a grade N48 neodymium magnet was introduced into a suspension of trypsinized cells that had been incubated with Ag–Magnetite or Au–Magnetite for 24 hours.

### Laser ablation ICP-MS

A NWR213 laser ablation system (ESI, Fremont, CA, USA) equipped with a two-volume cell was coupled to an ICP sector field mass spectrometer (Element XR, Thermo Fisher Scientific, Bremen, Germany). Details on operating parameters are given in the ESI (Tables S1 and S2†). The ICP-MS was synchronized with the LA unit in external triggering mode. Helium was used as carrier gas and argon was added before reaching the ICP torch using a Y-piece. The ICP-MS was tuned daily for maximum ion intensity and good signal stability (RSD < 5%), keeping the oxide ratio (ThO/Th) below 1% during ablation of a microscopic glass slide. An average day-to-day coefficient of variation of 10% for the ^137^Ba intensity was observed along the analyses. Representative areas of the cell sample with groups of individual fibroblast cells were ablated by continuous line scanning. The laser ablation parameters were optimized as described in detail by Drescher *et al.* ^49^ in order to achieve complete ablation of the cells, as well as high intensities at high spatial resolution. Potential polyatomic interferences on the iron isotopes were considered by measuring the gas blank before ablation and subtraction afterwards. The time-dependent ICP-MS intensities were exported to Origin 9.0 software (OriginLab Corporations, Northhampton, USA) to convert each raw data point to a single pixel in a color-coded surface plot. The time scale was transformed to a μm scale using the selected scan rate of 15 μm s^–1^. The integration of the intensities was performed using ImageJ software.^50^

### Quantification of the nanostructures in suspension and in cell extracts with ICP-MS

The uptake of nanoparticles by the cells was quantified using ICP-MS after digestion of the cells according to the following protocol: pellets of 5 × 10^5^ cells incubated with Ag–Magnetite for 24 h were digested with 600 μL 1 : 1 (v_cells_ : v_acid_) nitric acid (purified by sub-boiling distillation). Pellets of 5 × 10^5^ cells incubated with Au–Magnetite were digested with 600 μL *aqua regia* (450 μL hydrochloric acid and 150 μL nitric acid, purified by sub-boiling distillation).

To quantify the concentration of the nanoparticles in the cell incubation media by ICP-MS, 5 μL aliquots of the Ag–Magnetite and Au–Magnetite nanostructure suspensions were treated with 1 : 1 (v : v) nitric acid and *aqua regia*, respectively. The suspensions were homogenized directly before sampling using an analog vortex mixer (VWR International, Darmstadt, Germany). After addition of the respective acid, the samples were kept at room temperature overnight and afterwards were heated in a water bath at 70 °C for 2 hours. Blanks without cells and with cells, but without nanoparticles, were prepared in a similar manner. The digests were diluted with de-ionised water (18.2 MΩ cm, Milli-Q system from Millipore, Eschborn, Germany).

An iCAP Qc ICP mass spectrometer (Thermo Fisher Scientific, Bremen, Germany) was used for the determination of the Fe, Ag and Au concentrations. Due to the polyatomic interferences on the iron isotopes, the analysis was performed in kinetic energy discrimination (KED) mode using He as collision gas. The operating parameters are given in the ESI (Table S3†). The instrument was optimized daily for maximum sensitivity and an oxide ratio (CeO/Ce) below 3% in standard mode during nebulization of a multi-element tune solution. Afterwards the instrument was tuned in KED mode. For the calibration by standard addition, the samples were spiked at five different concentration levels with an iron, silver or gold solution prepared by dilution of the corresponding element stock solutions (Au: Plasma Standard Specpure, Alfa Aesar, Karlsruhe, Germany; Ag and Fe: ICP standard CertiPUR, Merck, Darmstadt). The measured element concentrations were converted to the number of Ag, Au or magnetite nanoparticles per liter or per cell using the density and the average nanoparticle diameters determined by transmission electron microscopy (Ag 53 nm, Au 29 nm, magnetite 5 nm) and assuming a spherical particle shape.

### SERS experiments with 3T3 fibroblast cells

SERS spectra of fibroblast cells incubated with Ag–Magnetite or Au–Magnetite for 3 and 24 hours were obtained with a microspectroscopic setup equipped with a liquid nitrogen-cooled charge-coupled device detector and a diode laser operating at 785 nm. The measurements were conducted using a 60× water immersion objective (Olympus, Hamburg, Germany) resulting in a laser spot size of 1.5 μm, and an excitation intensity of 2 × 10^5^ W cm^–2^. Spectra were recorded between 300 and 1800 cm^–1^ at a resolution of ∼5–8 cm^–1^. Cells were raster-scanned with a step size of 2 microns using an acquisition time of 1 s per spectrum. In the case of Au–Magnetite, seven cells were measured after 3 h-exposure and eight cells after 24 h-exposure. For the SERS experiments with Ag–Magnetite (3 h- and 24 h-exposure) five cells were investigated. The total number of Raman spectra (mapping points) from cells incubated with Ag–Magnetite for 3 h and 24 h was 1393 and 1448, respectively. The total number of Raman spectra from cells incubated with Au–Magnetite for 3 h and 24 h was 2406 and 1938, respectively. The spectra were frequency calibrated using a spectrum of a toluene-acetonitrile mixture, pre-processed, and screened for SERS signals. SERS spectra were extracted from the data sets and analyzed using MatLab (The MathWorks, Inc., Ismaning, Germany) and Cytospec (Cytospec, Inc., Berlin, Germany) software.

### Cryo soft X-ray nanotomography on intact 3T3 fibroblast cells

For X-ray microscopy, adherent mouse fibroblast cells were grown as a monolayer on Formvar-coated tomography grids, type HZB-2 (Gilder Grids, Lincolnshire, England) as described above. After 24 hours, the cells were exposed to Ag–Magnetite and Au–Magnetite for further 24 hours in cell culture medium (DMEM supplemented with 10% FCS). For vitrification, the X-ray tomography grids were washed three times with PBS buffer, blotted with filter paper and snap-frozen in a plunge freezer using liquid ethane. X-ray microscopy was performed at beamline U41-XM equipped with a cryostage at the electron storage ring BESSY II (Helmholtz-Zentrum Berlin für Materialien und Energie, Germany).^51–53^ Initial projection images of vitrified cells were collected at a tilt angle of 0°. Projection images of individual fibroblast cells were recorded at different angles with a typical tilt range of –60° to +60° in increments of 1° at a pixel size of 9.8 nm using a 25 nm zone plate objective. The exposure time for each tilt angle was varied between 2 s and 60 s depending on the sample thickness. First, the projection images were pre-processed by flat-field correction by averaging from 10 flat-field images (images without the specimen) obtained under the same experimental conditions and then normalized in order to correct for different beam currents and longer exposure times at higher tilt angles. For 3D imaging, alignment of the corrected tilt series and tomographic reconstruction were performed by the use of the software eTomo from IMOD.^54^ Images of a tilt series were aligned using the intracellular silver or gold nanoparticles as fiducial markers. A pixel binning of 2 × 2 was used for the tomographic reconstruction (projection images: no binning).

## Results

### Manipulation of cells with biocompatible, SERS-active Ag–Magnetite and Au–Magnetite composite nanostructures

**Fig. 1:**
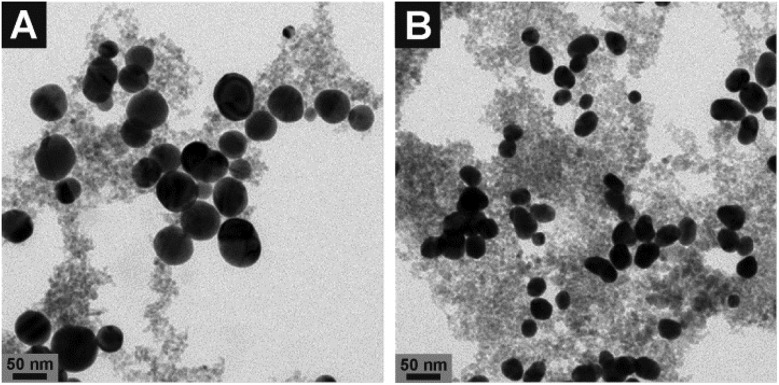
Transmission electron micrographs of (A) Ag–Magnetite and (B) Au–Magnetite. The images show spherical silver (A) and gold (B) nanoparticles (black spots) surrounded by smaller magnetite particles (grey spots). More images are shown in the ESI (Fig. S1C–F†).

The nanostructures Ag–Magnetite and Au–Magnetite were prepared by linking magnetite particles with Ag and Au nanoparticles, respectively, using (3-aminopropyl)triethoxysilane (APTES) according to Liang *et al.* ^46^ The size and morphology of the composite nanoparticles were characterized by transmission electron microscopy (TEM). The diameters of the silver nanoparticles are 53 ± 22 nm which are larger in size and show greater polydispersity (Fig. 1A and S1C, D†) than the gold nanoparticles (29 ± 5 nm, Fig. 1B and S1E, F†). The size of the magnetite nanoparticles is ∼5 nm. In accordance with the TEM micrographs, the absorbance spectra of the composite nanostructures show typical features of their respective silver and gold nanoparticles. The plasmon bands in the spectra do not change when the nanostructures are kept in typical cell culture medium (ESI, Fig. S1†), suggesting that the silver and gold nanoparticles are stabilized by their connection to the magnetite nanoparticles in the composite nanostructures. In the case of Au–Magnetite, a slight red-shift of the plasmon band (dashed line in Fig. S1B†) is observed. Even though the shift of the plasmon band is only indicative of a change in the refractive index around the gold or silver fraction, we assume that a biomolecular corona – responsible for the particle stability in physiological media during cellular uptake – forms around the whole composite nanoprobe, in accord with the observations made for magnetite nanoparticles.^29^

The SERS enhancement factors for the composite structures were estimated using the spectra of crystal violet.^47^ At an excitation wavelength of 785 nm, both Ag–Magnetite and Au–Magnetite show enhancement factors on the order of 10^3^ to 10^4^, in accord with the observation that many of the metal nanoparticles do not form aggregates but are kept separated from one another within their respective nanocomposite particles.^55,56^

An XTT cytotoxicity test demonstrates no toxicity effects of the composite nanoparticles on a 3T3 cell line (Fig. S2†). This is in good agreement with the previous work using the same gold and silver nanostructures separately.^57,58^ In contrast, similar gold–magnetite composite particles were observed to be toxic for bacteria growth.^59^ Based on their biocompatibility in our cell culture experiments we conclude that Ag–Magnetite and Au–Magnetite can be used as SERS nanoprobes in eukaryotic cells.

**Fig. 2:**
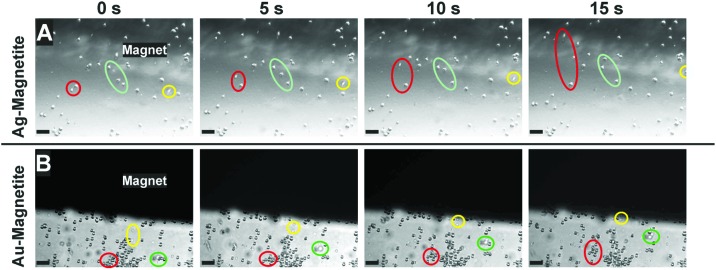
Microscopy images of suspended fibroblast cells after 24 h-exposure to (A) Ag–Magnetite and (B) Au–Magnetite. The series of micrographs are taken from a time span of 15 s. The motion of cell groups towards the magnet in the magnetic field is indicated by green, red and yellow marks. Scale bar: 100 μm.

To assess the possibility to manipulate cells containing the magnetic nanostructures, fibroblast cells incubated with Ag–Magnetite and Au–Magnetite for 24 hours were brought into an external magnetic field. After detaching the cells from their substrate by trypsin treatment, the cell suspensions were mixed thoroughly for 2 minutes, and a grade N48 neodymium magnet was placed in the suspensions. Fig. 2 displays the motion of fibroblast cells containing Ag–Magnetite (Fig. 2A) and Au–Magnetite (Fig. 2B) nanostructures in the magnetic field over a time span of 15 s. For both composite structures, displacement in the magnetic field is mainly observed not only for single cells (Fig. 2A and B: yellow labels) but also for groups of cells (Fig. 2B: green and red labels). Under the experimental conditions chosen, the velocity of the cells is on the order of 10 μm s^–1^. Relatively high variations (of a factor of two) were found between individual cells due to the fact that fibroblast cells naturally adhere again to the surface and subsequently detach again (for detailed information watch cells containing Ag–Magnetite in Movie S1† and Au–Magnetite in Movie S2†). The magnet-induced motion can be applied in microfluidic channels (for example, see Movie S3†). If the magnet is placed in the corner of a microfluidic channel that contains cells (*e.g.*, the lower right corner in Movie S3,† black spot), and the pumping is stopped, the flow of cells is reversed due to the magnetic force acting on the nanostructures contained in them. These results indicate that Ag–Magnetite and Au–Magnetite can be utilized for applications in magnetic cell separations, as well as in microfluidics.

### SERS spectra from cells

3T3 fibroblast cells were incubated with the Ag–Magnetite and Au–Magnetite nanocomposites for 3 hours and 24 hours, respectively. In total, 63 SERS spectra after incubation with Ag–Magnetite and 325 SERS spectra after incubation with Au–Magnetite were obtained. Considering the total number of mapping points in all the cells (see the Experimental section for details), ∼2–3% of the measured spectra of the cells show SERS signals after incubation of 3 hours with both kinds of composite structures. Interestingly, for Ag–Magnetite, the number of spectra with SERS signals does not change with an increase of the incubation time to 24 hours. In contrast, for Au–Magnetite, the portion of spectra with a SERS signal increased to ∼14% after 24 hours of incubation. The constant number or even an increase in SERS signals after 3 h and 24 h, respectively, can be regarded as an indicator of the high stability of the plasmonic properties of the silver and gold nanoparticles when they are linked to the magnetite nanoparticles, even in the harsh environment of the late endosomes and lysosomes that are typically probed after incubation times of a few hours.^60,61^ High stability of the signals is not necessarily the case for pure gold nanoparticles that tend to form large aggregates with relatively poor SERS performance after long incubation times,^56^ at least if the case of the particular fibroblast cell line used here is considered.

**Fig. 3:**
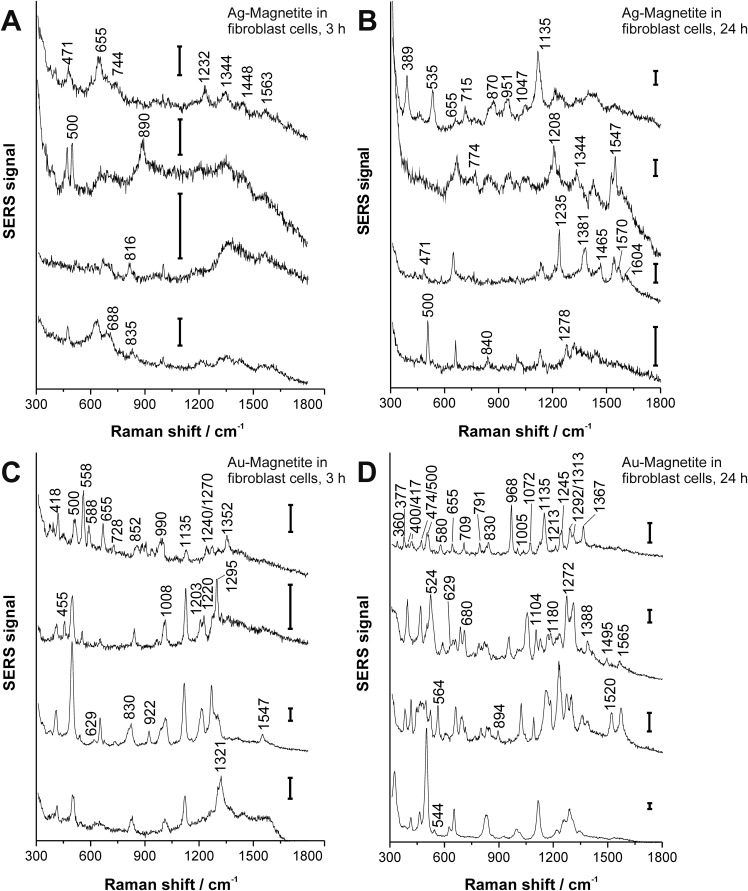
SERS spectra (representative examples extracted from mapping datasets) obtained from 3T3 fibroblast cells after 3 hours (A, C) and 24 hours (B, D) exposure to Ag–Magnetite (A, B) and Au–Magnetite (C, D). Excitation wavelength: 785 nm, excitation intensity: 1.9 × 10^5^ W cm^–2^, acquisition time: 1 s; scale bar: 100 cps.

The SERS spectra offer information about the composition of the chemical environment of the nanoparticles, specifically about the biomolecular corona formed in the cell culture medium, which is different for gold and silver nanoparticles,^62^ and which is observed by SERS to be modified in the cell depending on the incubation time.^56,63–66^ Fig. 3 shows typical spectra from the mapping datasets. Tentative assignments of prominent bands in these fingerprints are summarized in Table S4 of the ESI.† The number of bands in a SERS spectrum can be utilized to draw conclusions on the composition of the corona and on the properties of the plasmonic nanoaggregate(s) present in the focal volume.^56,62,63^ In the case of the Ag–Magnetite and Au–Magnetite composite nanostructures, we observe an increased number of bands in the SERS spectra for the longer incubation times. As an example, the amount of SERS spectra that contain at least six (random) signals increases in the data sets from ∼3% after 3 hours to 17% after 24 hours of incubation with the Ag–Magnetite nanostructures. After incubation with Au–Magnetite for 3 hours, 34% and after 24 hours, 55% of the SERS spectra show more than six bands. Based on our earlier observations made for pure silver and gold nanoparticles,^56,62^ we explain the higher number of signal-rich spectra obtained with the Au–Magnetite nanostructures by the larger variety of components in the nanoparticle corona (at least the part interacting with the gold fraction).

Many bands in the spectra obtained with the Ag–Magnetite nanostructures are caused by distinct cell medium components (*e.g.*, at 655 cm^–1^; *ν*(C–S) of cysteine^67^ and at 1208 cm^–1^ of the DMEM medium^58^), and resemble the cell spectra observed with pure silver nanoparticles.^58^ Different from the spectra of pure silver nanoparticles, after 24 hours of incubation (Fig. 3B), ∼10% of the spectra of the Ag–Magnetite composite nanoprobe contain also the contributions from intrinsic cellular molecules, *e.g.*, from amino acid mixtures and proteins. This is evidenced by the presence of bands assigned to the skeletal vibration of cysteine or proline/stretching (S–S) vibrations at 471 cm^–1^,^68^ by a pronounced band of the *ν*(S–S) stretching mode at ∼500 cm^–1^,^68^ or the C–NH_3_^+^ stretching and/or C_α1_H_2_ wagging vibrations at 1344 cm^–1^,^69^ as well as the 1547 cm^–1^ amide II band of proteins.^69^ The spectral fingerprints support that the composition of the biomolecular corona of the composite nanostructures is different from that of silver nanoparticles,^58^ which is possibly caused by the magnetite component of the nanoprobes that preferably interacts with other biomolecules than the silver parts would do in the absence of magnetite.^70^ We conclude that the protein corona formed on magnetite particles,^70–72^ can be observed in the composite nanostructures here due to the proximity of the magnetite part to gold and silver nanoparticles.

For Au–Magnetite, large qualitative variations between the spectra can be observed over time and within the cells, confirming the heterogeneity of the nanoparticles’ corona at different incubation times^73,74^ (Fig. 3C and D). This is in accord with previous investigations on uncoated gold nanoparticles in epithelial and fibroblast cells.^56,63^ Nonetheless, there are several frequently occurring combinations of signals, specifically those of the bands at 500 cm^–1^ (S–S stretching vibration), 629 cm^–1^ (C–S stretching vibration of cysteine, deformation vibration of COO^–^ and tyrosine side chain^67^), 830 cm^–1^ (tyrosine^68^) and 1135 cm^–1^ occurring from 15–40% (for examples see Fig. 3D).

**Fig. 4:**
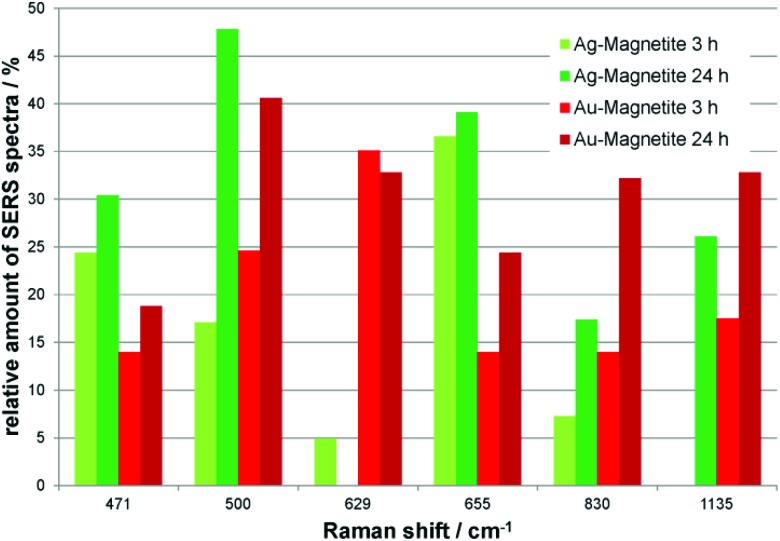
Relative amount of spectra containing SERS signals that occur in high abundance in fibroblast cells after incubation with Ag–Magnetite for 3 hours (light green) and 24 hours (dark green) and with Au–Magnetite for 3 hours (light red) and 24 hours (dark red).

Independent of the combined appearance, the frequency of typical signals that occur in many of the SERS spectra obtained with both Ag–Magnetite (Fig. 4, green bars) and Au–Magnetite (Fig. 4, red bars) was analyzed. For both structures, a general increase in the occurrence of several signals is visible after 24 hours of nanoprobe exposure. This is in good agreement with the increase of bands per spectrum discussed above (compare also Fig. 3). Especially for the bands at 500 cm^–1^ and at 830 cm^–1^ the occurrence is approximately doubled (compare the light with dark green and bright with dark red bars, respectively, in Fig. 4). Even though all bands analyzed in Fig. 4 are found with both the Ag–Magnetite and Au–Magnetite nanoprobes, their frequency differs between the silver and gold composite structures. For example, while the band at 629 cm^–1^ is often found in spectra measured from cells incubated with Au–Magnetite (Fig. 4, red bars), only a few spectra (<5%) with this band are observed with Ag–Magnetite (Fig. 4, green bars). *Vice versa*, there are many spectra measured with Ag–Magnetite that display signals at 471 cm^–1^ and 655 cm^–1^, the latter is found almost twice more often than with Au–Magnetite.

Interestingly, on comparing the bands that occur frequently in the composite Au–Magnetite nanoprobes with the spectra obtained with silica-coated gold nanoparticles reported previously, even in different cell types,^75^ we find many similarities in the spectra, indicating that both types of nanomaterials (silica and magnetite) must share parts of their biomolecular surface composition, differing from the surface of gold nanoparticles in a similar fashion.

In Fig. S3,† SERS mapping images of Ag–Magnetite and Au–Magnetite reveal the location of SERS active nanoaggregates inside the cell, based on important bands in the spectra that have been discussed above. The increased amount of spectra after 24 hours for both nanoprobes is clearly visible in the maps. The SERS maps show that the nanoprobes are distributed in the region of the cytoplasm but not in the nucleus. This is in agreement with the size of the composite nanostructures that does not permit penetration of the nuclear pore complex.^76,77^ These results are also in accord with our previous investigations on pure gold and silver nanoparticles of similar size^56,58^ as well as reports on other plasmonic–magnetic nanoparticles investigated in 3T3 fibroblast cells.^39^

### Intracellular quantification of the composite nanostructures

Magnetite-based composite structures can result in a different uptake compared to pure silver and gold nanoparticles, leading to changes in intracellular distribution and quantity due to their different physico-chemical properties, *e.g.*, size, shape and surface modification.^78–81^ To investigate the uptake of Ag–Magnetite and Au–Magnetite in individual 3T3 mouse fibroblast cells in comparison to non-linked silver and gold nanoparticles, laser ablation inductively coupled plasma mass spectrometry (LA-ICP-MS) micromapping experiments were conducted.

**Fig. 5:**
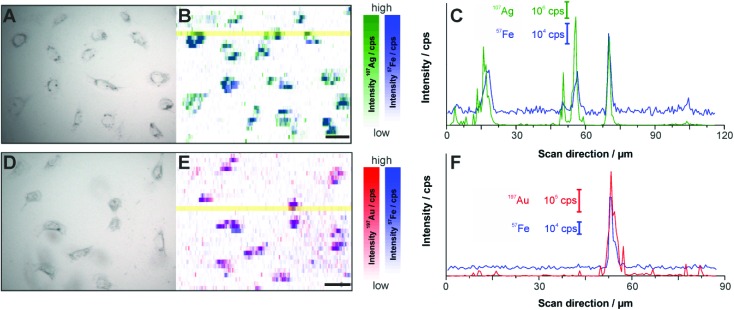
(A, B, D, E) LA-ICP-MS images of the ^107^Ag^+^ (green spots), ^197^Au^+^ (red spots) and ^57^Fe^+^ (blue spots) intensity distribution inside fibroblast cells after 24 h-exposure to Ag–Magnetite (B) and Au–Magnetite (E) and the corresponding bright field images (A, D). (C, F) Representative line scans of the cell regions marked yellow in B and E. (C) Intensity distribution of Ag–Magnetite and (F) of Au–Magnetite. Laser spot size: 15 μm, pixel size: 2.7 μm × 10 μm (Ag–Magnetite) and 2.8 μm × 10 μm (Au–Magnetite), line distance: 10 μm, scan speed: 15 μm s^–1^, repetition rate: 10 Hz, fluence: 0.3 J cm^–2^, scale bar: 50 μm.

Fig. 5 displays the LA-ICP-MS intensity maps and the corresponding bright field images of fixed cells exposed to Ag–Magnetite (Fig. 5A and B) and Au–Magnetite (Fig. 5D and E) for 24 hours. The ^107^Ag and ^57^Fe intensities (Fig. 5B) as well as the ^197^Au and ^57^Fe intensities (Fig. 5E and C) were measured simultaneously by continuous ablation line-by-line of an area comprising 10–20 cells. An ablation spot size of 15 μm is used in these experiments due to the lower sensitivity of ^57^Fe compared to ^107^Ag and ^197^Au caused by the low abundance of 2.1% ^57^Fe. By an optimized method using overlapping ablation spots we obtained a pixel size of 2.7 × 10 μm for Ag–Magnetite and 2.8 × 10 μm for Au–Magnetite in the scanning direction.^49,82^ The LA-ICP-MS intensities can be correlated with the local number of nanoparticles^49^ yielding the highest gold, silver and iron amount in the cytoplasm but not in the nucleus region, which is in accord with the observations made in the SERS maps (Fig. S3†). For both composites, the ^57^Fe signals are in spatial overlap with high intensities of ^107^Ag (Fig. 5B) and ^197^Au (Fig. 5E) which is also illustrated in single line scans (Fig. 5C and F). This confirms the stability of the composite nanoparticles during cellular uptake and processing.

**Fig. 6:**
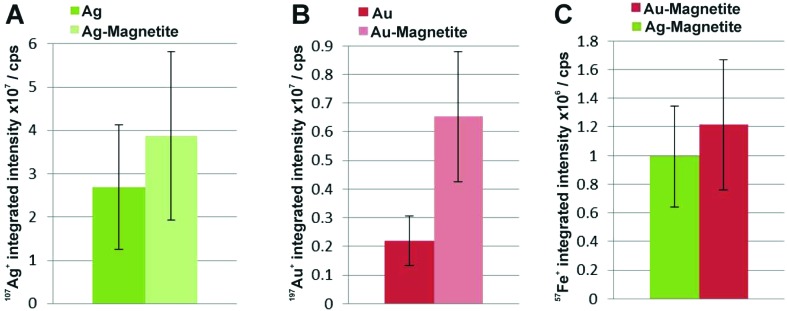
Integrated intensities of ^107^Ag^+^ (A), ^197^Au^+^ (B) and ^57^Fe^+^ (C) of single cells exposed to Ag–Magnetite and Au–Magnetite as well as non-linked silver and gold nanoparticles based on LA-ICP-MS data. The integrated intensities are given as the mean value of 10–20 fibroblast cells. The standard deviation reveals the cell-to-cell variability.

To compare the quantity of internalized Ag–Magnetite and Au–Magnetite with that of the non-linked silver and gold nanoparticles, we determined the integrated intensities per cell based on the LA-ICP-MS data. In Fig. 6, the integrated intensities of ^107^Ag^+^ (A), ^197^Au^+^ (B) and ^57^Fe^+^ (C) are given as the mean value of 10–20 fibroblast cells for each incubation condition. We found a slightly increased amount of silver (Fig. 6A) in 3T3 cells exposed to Ag–Magnetite compared to non-linked silver nanoparticles. Interestingly, ICP-MS analysis of digested composite structures of the nanoparticle suspensions proves nearly the same concentration of gold in the solutions of pure gold nanoparticles (3.6 × 10^–10^ M), and in the Au–Magnetite (3.3 × 10^–10^ M) solutions, the Ag–Magnetite nanoparticle solution contains one-third (7.7 × 10^–11^ M), of silver compared to the solution of pure silver nanoparticles (2.2 × 10^–10^ M). This indicates that, although the initial concentration of silver in the composite structure is lower, the cells take up more of the magnetite-linked silver nanostructures. Similar observation is made for fibroblasts after incubation with pure gold nanoparticles and Au–Magnetite, respectively. While in this case, the initial gold concentration in the incubation medium is similar, a three times higher amount of gold in the Au–Magnetite (Fig. 6B) is observed compared to the non-linked gold nanoparticles. We infer that, for both types of particles the uptake efficiency of the magnetite composites is approximately two to three times higher than that of the pure Au and Ag nanoparticles, which might be caused by an altered uptake mechanism due to the presence of magnetite. Magnetite nanoparticles are taken up by a two-step process^27^ and show a different behavior compared to silver and gold nanoparticles.^83^ The observation of a distinct biomolecular composition of the composite nanostructures by SERS discussed above also supports this. Furthermore, the amount of magnetite nanoparticles per fibroblast is similar for Ag–Magnetite and Au–Magnetite (Fig. 6C), supporting the fact that the presence of magnetite is an important determinant regarding the efficiency of uptake.

ICP-MS investigations of digested cells after exposure to composite structures for 24 hours reveal a total number of 1.8 × 10^3^ silver nanoparticles and 3.3 × 10^7^ magnetite nanoparticles per cell for Ag–Magnetite and 2.8 × 10^4^ gold nanoparticles and 4.0 × 10^7^ magnetite nanoparticles per cell for Au–Magnetite. An excess of several orders of magnitude is observed for the smaller magnetite nanoparticles. The similar amount of ^57^Fe^+^ found in both composite nanostructures in the analysis of digested cell pellets is in good agreement with the observations made by LA-ICP-MS mapping (Fig. 6C).

### Localization and aggregate morphology of composite nanoparticles

Cryo soft X-ray nanotomography with synchrotron radiation was performed to study the distribution and localization of the composite nanostructures, Ag–Magnetite and Au–Magnetite, in fibroblast cells after 24 hours of incubation. With this technique, ultrastructural 3D information can be obtained from whole eukaryotic cells that are adhering to their substrate, with a thickness of up to 10 μm and a 3D resolution down to 36 nm.^53,58^ Soft X-rays of a photon energy of 510 eV, in the so-called X-ray water window, experience a strong absorption by the organic material and by the inorganic composite nanoparticles.^84,85^ At this photon energy, the linear absorption coefficients (LACs) of iron (2.4 × 10^4^ cm^–1^) and carbon (4.4 × 10^4^ cm^–1^) are similar. In contrast, the LACs of the gold or silver particles are ten times higher (1.8 × 10^5^ cm^–1^ for silver and 2.2 × 10^5^ cm^–1^ for gold).

**Fig. 7:**
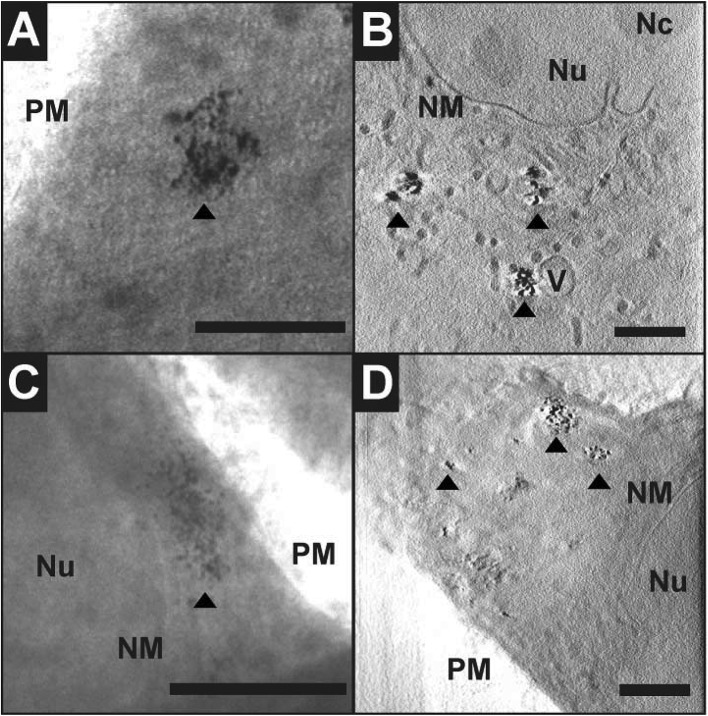
X-ray microscopy images (A, C) and tomographic slices (B, D) of vitrified 3T3 fibroblast cells after incubation with Ag–Magnetite (A, B) and Au–Magnetite (C, D) for 24 hours. The images were acquired with a 25 nm zone plate (9.8 nm pixel size). Arrows indicate the presence of composite nanoprobes. Scale bar: 1 μm. Abbreviations: Nu, nucleus; Nc, Nucleolus; NM, nuclear membrane; V, vesicle; PM, plasma membrane.

Fig. 7 displays the X-ray microscopy images from tilt series of cells incubated with Ag–Magnetite (Fig. 7A) and Au–Magnetite (Fig. 7C), respectively, and a slice of the respective tomographic reconstructions (Fig. 7B and D). The X-ray micrographs, as well as Movie S4 and S5† of microscopic tilt series in the supporting material show the presence of nanoaggregates in the cytoplasm but not in the nucleus for both types of nanocomposite materials, in agreement with the results of the SERS and LA-ICP-MS mapping experiments described above. Also TEM micrographs of magnetite nanostructures reported previously^86^ show that magnetite can easily enter the cell independent of the particle size and for a large range of particle sizes of 70–500 nm. The analysis of the projection images/tomographic slices based on the contrast of the plasmonic parts of the aggregates showed that the sizes of the Ag–Magnetite and Au–Magnetite nanostructures formed inside the endosomal structures of the cells are similar (264 ± 148 nm for Ag–Magnetite and 193 ± 133 nm for Au–Magnetite), with sizes covering a wide range between 50 nm and submicron size (Fig. S4†). This resembles the sizes of aggregates of unlinked silver and gold nanoparticles (253 ± 109 nm for silver nanoparticles and 223 ± 135 nm for gold nanoparticles). This is in contrast to other, pure magnetite nanoparticles, which form agglomerates of >600 nm in human breast cells.^87^

The tomographic slices (Fig. 7B and D) also display small, nm-sized gaps between the silver and gold nanoparticles for both composite substrates. Thus, based on our knowledge from electron microscopy of the nanostructures (Fig. 1A and B), we assume that these gaps correspond to the linked magnetite nanoparticles around the small silver and gold nanoparticles and nanoaggregates, and that the magnetite structures are not visible due to the magnetite's LAC being similar to that of the cellular ‘background’. Similar observations concerning spaces between the silver and gold nanoparticles were made for these plasmonic nanoparticles with silica shells that could be observed by cryo soft-XRT.^75^ In contrast, in non-linked silver and gold nanoparticles, very compact nanoaggregates without gaps are observed.^58^ The data show that the composite structures remain stable also in the harsh environment of the late endosomes and lysosomes, with the separation of the silver and gold nanoparticles, respectively, from one another by the magnetite nanoparticles being maintained. On the other hand, the results suggest that inside the cells the individual composite structures are present as aggregates and agglomerates, which would lead to more plasmonic nanoparticles coming into close proximity, thereby increasing the number of spots with high local fields. This is supported by the higher number of SERS spectra obtained for longer incubation, specifically after 24 hours (compare, *e.g.*, Fig. 4, Fig. S3†).

## Conclusions

In conclusion, the results presented here show that Ag–Magnetite and Au–Magnetite composite nanoprobes that have magnetic as well as plasmonic properties can be taken up by eukaryotic cells. The nanoprobes, both Ag–Magnetite and Au–Magnetite, are non-toxic and enable the mechanical manipulation of the cells, for example the transport in magnetic fields applied to microfluidic channels. As was revealed by the cryo soft-XRT data, the composite nanoparticles are contained in endosomes inside the cells, indicating their endocytic uptake. The nanostructures are stable even in the harsh environment of the late endosomes and lysosomes, suggesting their application as versatile optical and magnetic probes in the characterization of live cells. The high stability is specifically supported by the SERS data. The spectra provide information about the composition of the biomolecules at the nanoparticle surface. From the SERS spectra we infer that the surface composition of Ag–Magnetite and Au–Magnetite is different from that of pure gold or silver nanoparticles and influenced by the interaction of cellular biomolecules with the magnetite parts of the nanoprobes. LA-ICP-MS micromapping of intact cells and ICP-MS experiments on cellular extracts suggest that the endosomal uptake is determined by the magnetite component of the composite nanoprobes.
